# The Nucleus Accumbens: A Switchboard for Goal-Directed Behaviors

**DOI:** 10.1371/journal.pone.0005062

**Published:** 2009-04-07

**Authors:** Aaron J. Gruber, Rifat J. Hussain, Patricio O'Donnell

**Affiliations:** 1 Department of Anatomy & Neurobiology, University of Maryland School of Medicine, Baltimore, Maryland, United States of America; 2 Department of Psychiatry, University of Maryland School of Medicine, Baltimore, Maryland, United States of America; 3 Center for Neuropharmacology and Neuroscience, Albany Medical College, Albany, New York, United States of America; Minnesota State University Mankato, United States of America

## Abstract

Reward intake optimization requires a balance between exploiting known sources of rewards and exploring for new sources. The prefrontal cortex (PFC) and associated basal ganglia circuits are likely candidates as neural structures responsible for such balance, while the hippocampus may be responsible for spatial/contextual information. Although studies have assessed interactions between hippocampus and PFC, and between hippocampus and the nucleus accumbens (NA), it is not known whether 3-way interactions among these structures vary under different behavioral conditions. Here, we investigated these interactions with multichannel recordings while rats explored an operant chamber and while they performed a learned lever-pressing task for reward in the same chamber shortly afterward. Neural firing and local field potentials in the NA core synchronized with hippocampal activity during spatial exploration, but during lever pressing they instead synchronized more strongly with the PFC. The latter is likely due to transient drive of NA neurons by bursting prefrontal activation, as *in vivo* intracellular recordings in anesthetized rats revealed that NA up states can transiently synchronize with spontaneous PFC activity and PFC stimulation with a bursting pattern reliably evoked up states in NA neurons. Thus, the ability to switch synchronization in a task-dependent manner indicates that the NA core can dynamically select its inputs to suit environmental demands, thereby contributing to decision-making, a function that was thought to primarily depend on the PFC.

## Introduction

Studies in primates and rodents identify frontal cortical areas as critically involved in decision making [1], [2], [3], [4]. These regions control behavior by virtue of their projections to other cortical and subcortical areas, most notably the basal ganglia [5]. The NA, a component of the ventral striatum in basal ganglia circuits, plays a central role in motivated, goal-directed behaviors by integrating limbic and prefrontal cortical information [5], [6] and has been described as an interface between limbic and motor systems [7], [8]. The role of the NA in guiding behavior may depend on the composition of active neural assemblies determined by the state of afferent inputs [9]. Indeed, the NA receives dense glutamatergic projections from the medial PFC [10] and ventral hippocampus (VH) [11], and the interactions between these inputs determine action potential firing in the NA [12]. NA neurons can become entrained to hippocampal theta rhythms (4–8 Hz) in awake rats [13], and NA firing follows VH firing with a delay consistent with monosynaptic inputs [14]. Furthermore, the coordinated theta activity in the hippocampus and striatum can influence interactions during learned behaviors [15]. Such behaviors also activate the PFC, and simultaneous recordings in the hippocampus and PFC revealed a strong coupling between both structures in the theta range, as well as correlated cell firing during spatial working memory tasks [16]. As the medial PFC is involved in set shifting [17], conflict resolution [3], tracking errors [18], and developing stimulus-outcome associations [19], and its task-related neural responses often occur as phasic bursts [20], [21] it is likely to exert brief yet strong influence on the NA during decision making. Thus, a three-way interaction among the PFC, VH and NA may be critical for switching behaviors. To address this issue, we recorded electrical activity simultaneously in all three structures during two different behavioral conditions in the same session; first, when the rats were actively exploring the cage, and a few minutes later when lever-pressing for a sucrose reward. As projections from the medial PFC and VH converge on individual NA medium spiny neurons (MSNs) [12], [22], it is possible that the dominant area during a given behavioral condition determines the pattern of NA firing. Here we tested whether the synchrony of the NA with the PFC and VH differs between a VH-dominated condition (spatial exploration) and a condition with brief but strong PFC activation (lever press for reward).

## Methods

All experiments were performed according to United States Public Health Service *Guide for the Care and Use of Laboratory Animals* and were approved by the Albany Medical College Institutional Animal Care and Use Committee and the University of Maryland School of Medicine Institutional Animal Care and Use Committee.

### Multichannel recordings in awake animals

Male Sprague Dawley rats (n = 6) weighing between 300 and 375 g at the time of recording were obtained from Charles River Laboratories (Wilmington, MA). Each rat was housed individually in a reversed 12 hr dark/light cycle (on: 8 PM; off: 8 AM), so recordings were conducted during the animals' active phase. Water availability was restricted to 15 ml/day for 2–3 days prior and during the training, but rats had full access to food. Animals were maintained at no less than 85% of the pre-surgical body weight. Training sessions (30 min or 65 sucrose deliveries/day) were conducted in a Plexiglas chamber enclosed in a sound attenuating box (Med Associates, St. Albans, VT) provided with a fluid receptacle positioned 2 cm from the bottom of the chamber and 2 retractable levers on either side of the receptacle. Cue lights were 4 cm above the levers. Animals were trained on a fixed ratio 1 (FR1) to press a lever for sucrose reinforcement (10% solution; 50 µl/press). The training session started with turning on the house light and background white noise. Each trial started with the cue light and lever extension simultaneously. After a press, the lever retracted and sucrose was delivered. The task was not designed to assess working memory, but to ensure the rat was actively exploiting reward availability. There was a 20 sec delay between trials. Experimental events were controlled and recorded using Med PC software from Med Associates. Once trained (>2.7 presses/min), rats had full access to water for 3–4 days prior to surgery.

Rats were anesthetized with ketamine (70 mg/kg, i.m.) and xylazine (7 mg/kg, i.p.), and placed on a stereotaxic apparatus with non-puncturing ear bars (David Kopf instruments, Tujunga, CA). Body temperature was maintained at 36–38°C using a heating pad and monitored with a rectal temperature probe (Fine Science Tools, Foster city, CA). Anesthesia was maintained by subsequent ketamine (20 mg/kg) and xylazine (2 mg/kg) injections every 60–90 min. Bupivacaine (0.25%) was applied subcutaneously before any skin incision was made. Small holes were drilled unilaterally in the skull to fit linear 1×8 or square 2×3×3 microelectrode arrays (NB Labs, Denison, TX). Six rats had microelectrodes implanted in the PFC, NA and VH (Figure S2). The coordinates [23] for the NA were: 1.4–1.6 mm rostral to bregma (AP), 0.5–2.5 mm from midline (ML) and 7.0–7.5 mm from skull surface (DV). For the VH, they were (in mm): −4.8–5.6 AP, 3.5 ML and 7.0–8.0 DV; for the PFC, they were 2.8–3.5 AP, 0.5–1 ML and 4–4.5 DV. Five to six additional holes were made for ground wires and screws to anchor all the implants with dental cement. At the end of the surgery each rat received diazepam (5 mg/kg, ip) and enrofloxazine (2.3 mg/kg, ip), as well as saline (12–15 ml, sc). Antibiotic ointment was applied topically around the implant for 2–3 days. Rats were given 2 weeks for recovery before starting the recording sessions.

Recording sessions (4–6 per rat) were comprised of an initial 5–10 minute exploration period in which the chamber light was off and the lever was retracted. Rats typically explored the chamber, walking around and frequently attending to the wall opening from which the lever would later extend. Following the exploration period, the FR(1) lever pressing task was initiated. During both phases of the session, we simultaneously recorded field potentials and the activity of single units using a multichannel acquisition system (Plexon Inc., Dallas, TX). For single units, gains were set for each channel prior to the exploration phase. During sessions, 800 µs-long waveforms of all electrical events that were more than 3 standard deviations from background noise were saved to a hard drive for offline analysis. The times of behavioral events (lever extension, lever press, sucrose delivery and reward entry) were also recorded with the same system. We determined the times at which rats were facing the retracted lever port during the exploration phase by visual inspection of a recorded video tape of rats in the chamber. Principal component analysis (Offline Sorter, Plexon) of saved waveforms was used to isolate firing events originating from single units and reject artifacts and events originating from other units. Units were excluded from the study if their activity rates or waveform amplitudes were not stable during the session, or if a 2 ms refractory period was not observed. The activity of single units was analyzed with Neuroexplorer (Plexon). To determine the correlation among PFC-NA and VH-NA, cross-correlograms of single unit activity were generated for exploration of the lever port and for bar press (±2 s window and 10 ms bin size). Values of peak cross-correlations were normalized by the peak cross-correlation obtained by shuffling the spike timing of the source data. Each unit was used in only one PFC-NA and VH-NA comparison. A one-tailed paired t test was used to test the null hypothesis that normalized peak cross-correlations did not increase during the instrumental behavior. Data from only sessions in which rats approached the retracted lever at least 10 times during the exploration phase, and in which rats bar pressed at least 20 times were included in our analysis. Power analysis was computed using a commercial software package (Power and Precision, Biostat Inc.) to test whether the number of paired recordings was sufficient to substantiate the outcome of statistical hypothesis testing.

Local Field Potentials (LFP) were recorded simultaneously with single unit activity from the same microwire arrays. Four microwires with confirmed histology were selected from NA core, PFC and VH in every rat, and 2 microwires with confirmed histology were selected from NA shell in 2 rats. Field potential data were acquired at 1 kHz using a National Instruments A/D data acquisition card (NIDAQ), and the data were analyzed offline. For each microwire, 2–3 different recording sessions were analyzed with open source algorithms (http://chronux.org) and custom algorithms implemented in Matlab to explore their time-frequency properties. The multitaper method was used to window LFPs and the Fast Fourier Transform (FFT) was used to estimate spectral power. Spectrograms and time-dependent changes in cross-spectral densities were constructed by plotting spectral power of consecutive sliding windows of constant duration (1 sec). Larger windows (4 sec) centered on behavioral events were used to compute spectral power of LFP over the duration of the lever pressing and exploratory behaviors. Coherence between similar-frequency peaks in the individual spectra was calculated using the Chronux toolbox, as described in previous work by the toolbox's authors [24], by standardizing the cross-amplitude values. Cross-spectral densities were squared and divided by the product of spectral densities of each recording. The result was interpreted as a squared correlation coefficient (r^2^) for statistical purposes. High coherence values (>0.75) were taken as indicators of oscillatory activity at that particular frequency being synchronized. To explore contextual shifts in processing among structures over a longer time epoch, additional analysis was performed over 20 second intervals centered on lever pressed and lever port exploration. Power spectral densities for these longer intervals were computed by FFT using a Hamming window (implemented in Statistica) as previously reported [25], [26], and summed over the range of 1–4 Hz (delta), 4–8 Hz (theta), 8–14 Hz (alpha), 14–30 Hz (beta) and 30–50 Hz (gamma) to compare spectral bands during exploration and bar press.

At the end of the final recording session, rats were deeply anesthetized with sodium pentobarbital (100 mg/kg). In order to mark the recording sites, 10–20 µA current was passed for 5–10 sec in selected wires to deposit iron. Rats were transcardially perfused with ice-cold saline followed by formalin solution, and brains were removed and post fixed in formalin for at least 24 hr. Serial 40–50 µm coronal sections were cut through the PFC, NA, and VH using a freezing microtome, and mounted on gelatin-coated slides. All sections were Nissl-stained, counterstained with Prussian blue (5% potassium ferricyanide in 10% HCl) for electrode tip placement, cover slipped in Permount and examined on an Olympus microscope (CH30, Tokyo, Japan) using the Paxinos and Watson atlas [23] for reference. Only data from wires with confirmed placement were used in the analyses.

### *In vivo* intracellular recordings in anesthetized rats

Intracellular recordings were conducted in 13 male Sprague Dawley rats (Charles River Laboratories, Wilmington, MA) weighing 260–430 g. Rats were initially anesthetized with chloral hydrate (400 mg/kg, i.p.), and subsequent anesthesia was maintained via constant infusion of chloral hydrate (20–30 mg/h, i.p.) using a syringe pump (Bioanalytical Systems, West Lafayette, IN). Body temperature was maintained at 36–38°C using a heating pad and monitored with a rectal temperature probe (Fine Science Tools). Rats were placed in a stereotaxic apparatus (David Kopf), and an array of electrodes was implanted in the right prelimbic cortex (centroid of array tips with respect to bregma: 3.2 mm rostral and 0.6 mm lateral; depth: 4.3 mm ventral from skull; 30° angle toward midline). Electrode arrays were constructed with commercially available tungsten electrodes (WPI Inc., Sarasota, FL) or with 115 µm Teflon-coated tungsten wire (A-M Systems Inc., Carlsborg, WA). Electrode pairs were connected to a switchbox that allowed for the recording of cortical field potentials or delivery of electrical stimulation. Field potentials were amplified 1,000 times (DP-301 differential amplifier, Warner Instrument Corp., Hamden, CT), passed through a noise eliminator (Humbug, Quest Scientific Instr., Vancouver, Canada), digitized (Digidata 1322A, Axon Instruments, Union City, CA), band-pass filtered at 0.1–5 kHz, sampled at 10 kHz and saved to a hard drive using Axoscope software.

Intracellular recording electrodes were pulled from 1 mm (o.d.) borosilicate glass tubing (WPI) to a resistance of 40–100 MΩ with a P-97 Flaming-Brown microelectrode puller (Sutter Instruments, Novato, CA). Recording electrodes were filled with 2% Neurobiotin (Vector Laboratories, Burlingame, CA) in 2 M potassium acetate and lowered into the right NA with a hydraulic microdrive (Trent Wells, Coulterville, CA) within the following range of coordinates: 1.3–1.7 mm rostral to bregma, 1.2–1.4 mm lateral to the midline, and 5.5–7.5 mm ventral from the cortical surface. Intracellular signals from the recording electrode were amplified (IR-283; Neurodata, Delaware Water Gap, PA), low pass filtered at 2 kHz (FLA-01, Cygnus Tech. Inc., Delaware Water Gap, PA), digitized (Digidata 1322A, Axon Instruments), sampled at 10 KHz using Axoscope (Axon Instruments) and stored on a PC. Once a cell was impaled and the membrane potential stabilized, spontaneous fluctuations of membrane potential were recorded simultaneously with cortical field potentials, one at a time, from different cortical electrodes. The cortical electrode giving the best relationship with the activity of the recorded MSN was used for analysis. In order for a cell to be included in the present data set, its resting membrane potential had to be more negative than −60 mV, its action potential amplitude greater than 40 mV measured from threshold, and input resistance greater than 35 MΩ.

Following electrophysiological recordings, Neurobiotin was injected into cells by passing positive current (0.5–1.0 nA, 200 ms pulses at 2 Hz) for 5–15 minutes through the recording electrode. At the completion of the experiments, rats were euthanized with an overdose of pentobarbital (100 mg/kg) and transcardially perfused with cold saline followed by cold 4% paraformaldehyde. Brains were postfixed in 4% paraformaldehyde for at least 24 h before being transferred to a solution of 30% sucrose in 0.1 M phosphate buffer for cryoprotection. Sections were cut (30–40 µm) using a freezing microtome and placed in phosphate buffer. Sections through the PFC were mounted on gelatin-coated slides and Nissl stained to verify placement of electrodes. Sections through the NA were processed for visualization of Neurobiotin-filled cells.

Traces of simultaneously recorded cortical LFP and intracellular NA neuron membrane potential were analyzed using native and custom-built functions implemented in Matlab (Mathworks, Natick, MA). First, up and down states were detected with a deadband threshold algorithm in which up states were defined as times in which the membrane potential exceeded an upper threshold and remained above a second lower threshold for at least 200 ms. The threshold varied from cell to cell, but was generally set near the middle of the voltage range between up and down states. Sliding window cross-covariance was computed for these time traces with a ±200 ms lag window and 5 ms time steps. Next, mean cross-covariance was computed over all transitions to the up state and quantified by the distribution of magnitude and lag of their peaks. Statistical significance was determined by comparing distributions of those values with the distribution of cross-covariance computed in the same manner with surrogate traces. Surrogate trace sets were comprised of unaltered intracellular traces and randomized cortical field traces constructed by 1) computing the FFT of the signal, 2) randomizing the phases of the FFT, and 3) using inverse FFT to reconstruct a time series with the same spectral magnitudes but shuffled phases from the original cortical field trace. The Kolmogorov-Smirnov test was used to determine significance of peak and zero-lag cross covariance values of data as compared to that of 50 iterations with randomized field traces.

## Results

**Figure 1:**
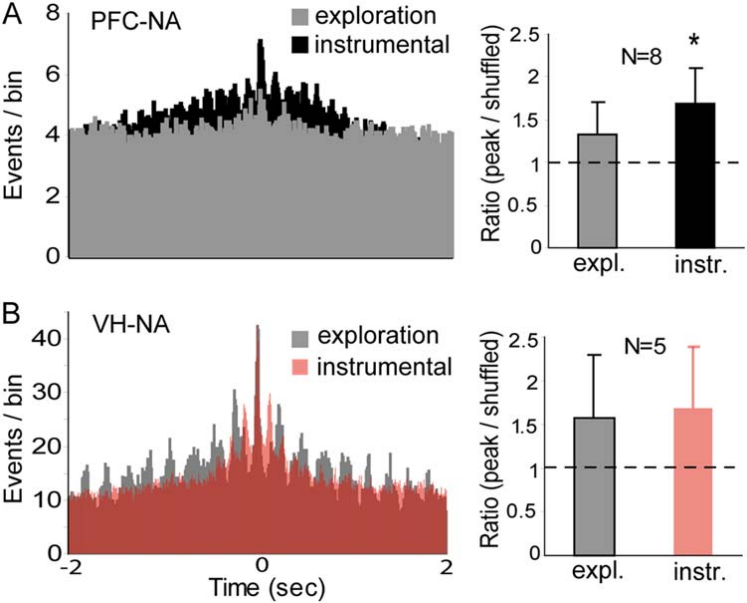
PFC-NA unit correlation is strengthened during bar press for a natural reward. (A) Representative cross-correlograms of a NA-PFC neuron pair (referenced to PFC firing at time 0) when the animal was exploring (gray) and during bar pressing for sucrose (black). Right: bar graphs showing the ratio between the crosscorrelogram peak and a similar analysis of shuffled recordings from the same pairs for both behavioral conditions. Mean±SD; * p<0.05 by paired t test. (B) Similar cross-correlation of a representative VH-NA pair during exploration (gray) and bar pressing (red). No differences were observed in these cases.

We recorded single-unit activity simultaneously in the NA, VH and PFC in awake, freely moving rats using chronically implanted electrode arrays. Recordings were carried out during two different behavioral conditions in the same session: first, when rats were exploring the test chamber during the initial 5–10 minutes of recording while the lever was retracted, and then during an immediately following period of 10–20 minutes when rats were required to press the extended lever in order to obtain a natural reward (sucrose). A visual cue and the lever extension indicated availability of the reward, and rats typically pressed the lever within one second. NA (49/60), PFC (30/34) and VH (8/15) neurons exhibited phasic changes in firing relative to the lever press. All the VH neurons that responded showed an increase in firing following the lever press; in the NA and PFC, units exhibited increases or decreases in firing associated with the lever press or with the reward delivery, as previously reported [20], [27] (Figure S1, Table S1). We then assessed whether synchronization between neurons across brain regions changed with the behavioral state by constructing cross-correlograms of neural responses during lever presses and during epochs of the exploration phase in which rats were moving toward and making contact with the lever port. These epochs of the exploration phase were confirmed by video recordings of rats in the chamber, and were chosen so that spatial information was similar in both behavioral conditions. For this analysis, we included units from the three animals in which single unit responses were recorded in all three regions simultaneously. Each NA unit used in a HC-NA comparison was also used in a PFC-NA comparison so as to provide a within-subject control. Likewise, data for the two behavioral conditions are from the same session, providing a within-session control. All NA-PFC pairs tested (n = 8) showed enhanced correlation during the bar press compared to the exploratory period (Figure 1A). The strength of the correlation was calculated by determining the ratio between crosscorrelogram peaks and shuffled crosscorrelograms. For NA-PFC pairs, this ratio was 1.33±0.36 during the exploratory stage and increased to 1.67±0.40 during bar press (n = 8; p = 0.014, paired t test). The correlation between NA and VH neurons did not differ between exploration and the instrumental task (1.60±0.72 during exploration; 1.70±0.70 during bar press; n = 5; p = 0.37, paired t test; Figure 1B). Power analysis revealed 77% confidence for the NA-PFC relationship and 92% confidence for the HC-NA relationship, indicating robust power despite the limited number of simultaneous single-unit recordings. Thus, lever-pressing behavior was associated with a stronger PFC-NA correlation in neuronal firing than spatial exploration.

**Figure 2:**
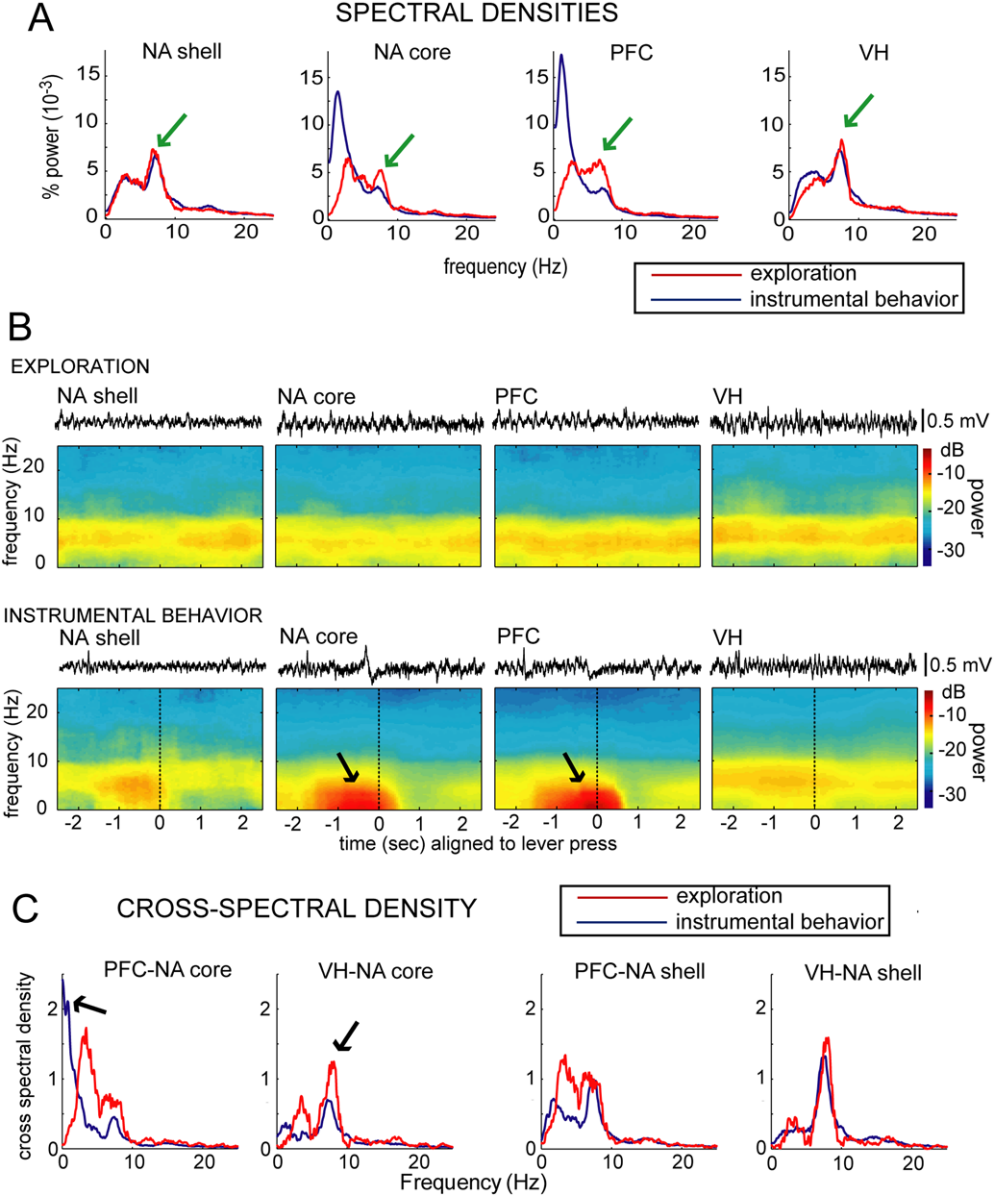
Dominant frequencies in the VH, NA and PFC field potentials differ between exploration and goal-directed behavior. (A) Normalized spectral densities in the NA shell, NA core, PFC and VH obtained from simultaneously recorded epochs (4 seconds) in which the animals were exploring the cage (red line). The epochs were selected to match the location and body orientation of the operant task. The blue line represents the normalized spectral densities for the same four locations but during 4 second epochs in which the rats were lever-pressing for sucrose (2 seconds prior and after the lever press). The graphs were constructed with data from 6 sessions in 5 rats for the NA core, and 2 sessions in 2 rats for the NA shell (all of them with simultaneous recordings in the PFC and VH). Strong theta peaks are evident in all regions during exploration (green arrows), but they are lost in the NA core and PFC during the instrumental behavior. An increase in delta activity can be observed instead. (B) Pseudocolor plots of relative spectral power in the NA shell, NA core, PFC and VH during a 5-second epoch in which rats were exploring (top) and during a 5-second epoch centered on the lever press when the animals were engaged in instrumental behavior (bottom). The LFP traces of one of the epochs included in the analyses are shown above each box. Event-triggered and exploration spectrograms were constructed from one session from each animal and the display is the averaged data of all animals, revealing a strong theta oscillation during exploration, which weakens in the NA core and PFC (but not in the NA shell and VH) during lever-pressing. The NA core and PFC show instead strong activity in the delta range (arrows), which are driven by slow deflections that can be observed in the traces above. (D) Cross-spectral densities were calculated to determine coherence between similar frequency peaks in LFP obtained simultaneously from different brain regions during exploration and instrumental behavior. The two leftward panels illustrate representative pairings of PFC and NA core, and VH and NA core while the rat was exploring (red line), revealing a high coherence in the theta range between VH and NA core (arrow in second panel from left). The blue line in both panels are cross-spectral densities in the same pairs when the rat was bar pressing for sucrose in the same session, showing a peak in the delta range between NA core and PFC (arrow in left panel). The two rightward panels illustrate cross-spectral densities between the NA shell and PFC and VH in the same rat and session. A strong theta peak is present in the shell-VH cross-spectrum independently of the behavioral condition.

**Figure 3:**
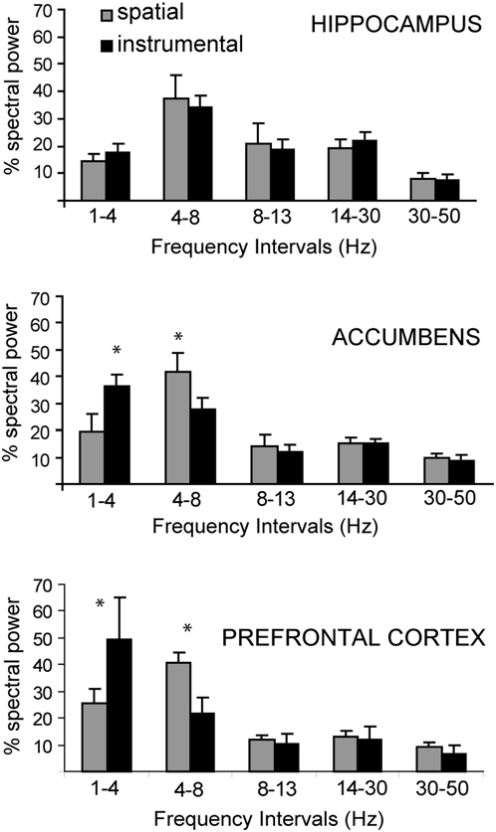
Weight of spectral bands during spatial exploration and goal-directed behavior. Bar graphs depicting summed power for the 1–4 Hz (delta), 4–8 Hz (theta), 8–14 Hz (alpha), 14–30 (beta), and 30–50 (gamma) bands. Gray bars show the weight of each band during spatial exploration and black bars represent band weight during goal-directed behavior. Top to bottom graphs illustrate spectral bands from all accumbens core, hippocampal, and PFC recordings.

Local field potentials (LFPs) were recorded simultaneously from the same wires that yielded single-unit data. Frequency components in this measure of population activity were determined using the Fast Fourier Transform. During epochs of exploration, this analysis revealed a dominant 7–8 Hz (theta) peak in all regions (green arrows in Figure 2A). Theta oscillations are found in different mammalian brain regions but most prominently in the hippocampus [28] and are associated with complex behaviors like spatial exploration and memory functions [29], [30]. During the operant task, the VH and NA shell still exhibited a dominant theta peak, but the PFC and NA core did not. Instead, the PFC and NA core exhibited dominant rhythms in the delta range (1–4 Hz; blue line in Figure 2*A*; statistical comparisons shown in Figure 3). A multi-taper spectral analysis [15] allowed us to calculate spectral density over short time intervals, revealing that the increase in delta power in the NA core and PFC occurred during the 1–2 second interval between the cue and lever press (arrows in Figure 2*B*). These low frequency events are unlikely to be artifacts of locomotion as they were not detected in the nearby NA shell electrodes. Thus, low-frequency delta events in the medial PFC and NA core are important elements in the corticoaccumbens system during instrumental behavior, but not during spatial exploration.

We also assessed synchronization of LFP activity among these regions in both components of the recording session. During spatial exploration, a strong correlation existed between the PFC, NA (both core and shell), and the VH for simultaneous peaks in the theta range. During the instrumental component, a correlation was detected in the delta band between the medial PFC and NA core. Co-spectral density of simultaneously recorded LFPs revealed simultaneous theta peaks in the NA and VH during exploration, not lever-pressing, and coincident NA-PFC delta peaks during the instrumental behavior (Figure 2*C*). Coherence was calculated from these plots to obtain a statistical measure of correlation (r^2^). During exploratory activity, NA core and VH LFPs exhibited high coherence at 7.8±0.5 Hz (r^2^ = 0.94±0.04; 18 pairs in 6 rats). Such correlation between VH and NA theta peaks could not be observed during bar pressing for sucrose (Figure 2C), suggesting that hippocampal control of NA activity is weaker during goal-directed behaviors. The correlation between NA and PFC spectral peaks exhibited a different pattern. NA core-PFC LFPs exhibited high coherence during bar press for sucrose (2.6±0.8 Hz; r^2^ = 0.94±0.03; 20 pairs in 6 rats). As the PFC-NA and VH-NA connections are not reciprocal (both areas send heavy monosynaptic projections to the NA), the coherence data suggest that NA core follows VH activity during spatial exploration, but follows PFC activity during an operant task, even in the presence of similar spatial information. In the two rats with recordings in the NA shell, no increase in delta activity was seen during the instrumental component (Figure 2*B*) and the spectra remained identical for both conditions (Figure 2*A*). Thus, the link between the NA core and PFC changes with the behavioral conditions. During reward-seeking behavior, strong PFC activation may be able to override the tight NA correlation with VH theta rhythms that characterized the exploratory period.

**Figure 4:**
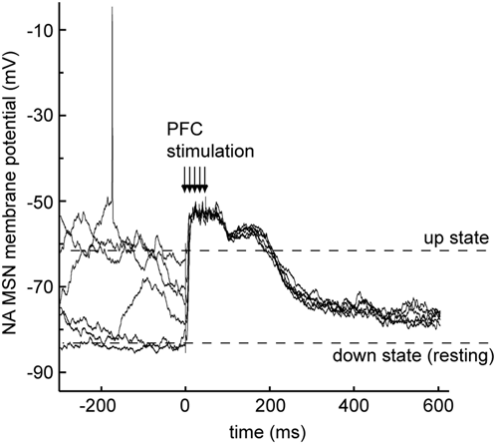
PFC stimulation with trains of pulses evokes persistent depolarizations in NA neurons. Overlay of six traces obtained from a NA neuron during *in vivo* intracellular recording from an anesthetized rat, showing the membrane potential responses to stimulating the PFC with a train of 5 pulses at 50 Hz (arrows indicate the stimulation times; stimulus artifacts were removed for clarity). The traces were selected to display stimuli delivered during both up and down states, and in either case a sustained depolarization was observed.

Does the transient PFC-NA core synchronization reflect the ability of brief epochs of PFC activity to drive up states in NA MSNs? We tested this possibility by stimulating the PFC with a bursty pattern similar to what observed in awake rats during goal-directed behavior [20]. Unlike single-pulse stimulation [12], electrical stimulation of PFC with trains of pulses (5 pulses at 50 Hz) evoked sustained depolarizations similar to up states in NA core MSNs of anesthetized rats (n = 5; Figure 4). This suggests that high frequency PFC firing, such as what occurs during instrumental behavior, could exert a brief but powerful influence on the membrane potential of NA neurons.

**Figure 5:**
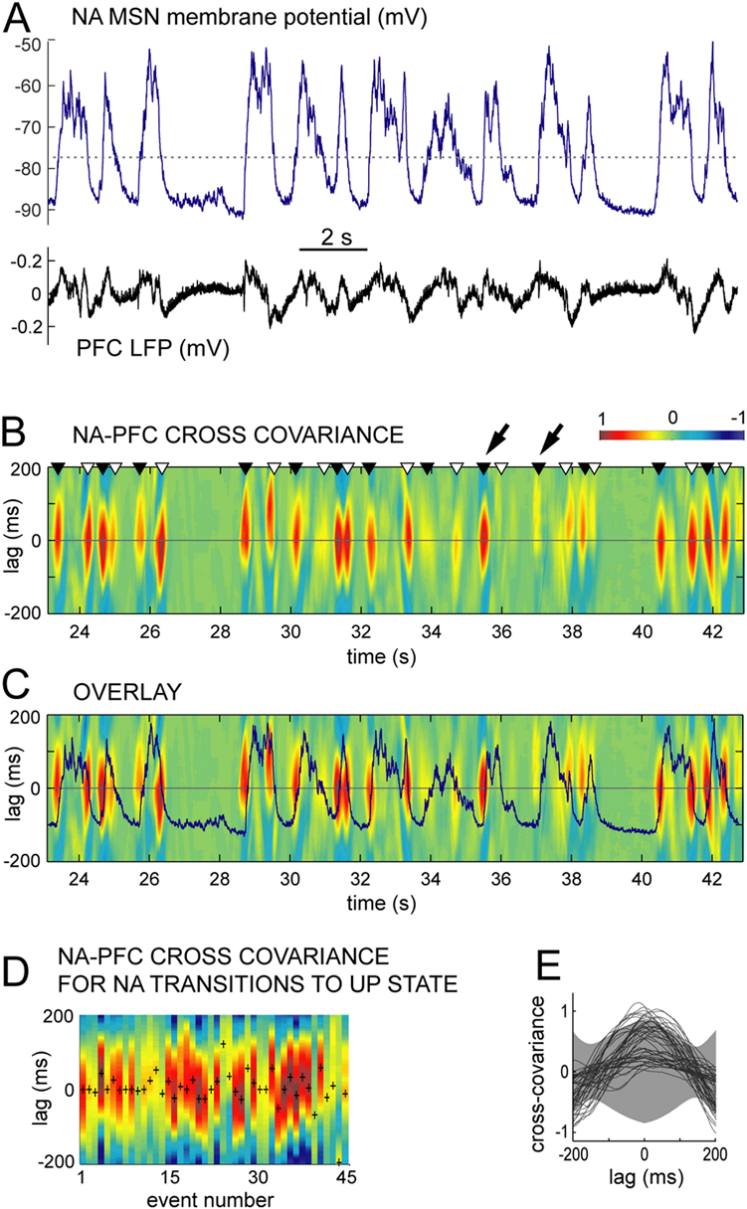
Cross-covariance analysis of intracellular NA neuron membrane potential and PFC LFP in anesthetized rats. (A) Simultaneous recording of intracellular membrane potential of a NA medium spiny neuron (MSN; top) and PFC field potential (LFP; bottom) showing spontaneous oscillations typical of an anesthetized rat. Transitions of MSN membrane potential between a hyperpolarized down state and depolarized up states are detected with a threshold (dotted line). (B) Pseudocolor plot of the cross covariance of these traces with a ±200 ms time lag window (ordinate). MSN membrane potential transitions from down to up states are indicated by dark triangles, and up-to-down transitions with white triangles. Oblique arrows point to two consecutive up state onsets showing high covariance (left) and no covariance (right) with PFC LFP. (C) Overlay of the cross covariance plot in B and the MSN membrane potential trace in A showing the high covariance epochs to correspond to state transitions. (D) Cross covariance at successive down-to-up state transitions from data in A, showing that both the magnitude and lag of the peak cross-covariance (indicated by ‘+’) vary in time. Transitions that co-vary with PFC LFP are interspersed with those that do not co-vary. (E) Cross covariance in B plotted in time and superimposed with mean±standard deviation of cross covariance computed from randomized versions of the traces in A (gray region). Cross-covariance near lag = 0 shows the data clustered in two populations: a significantly covariant set of events and others with almost no covariance.

The data predict that variations in PFC activity could affect active periods in the membrane potential of NA MSNs (up states). Electrical VH stimulation evokes up states in NA neurons [12] and fornix or VH lesions eliminate these up states [12], [31], arguing for a strong hippocampal control of NA up states, but the data shown in Figure 4 suggest that PFC inputs may also exert at least a transient control over NA up states. We therefore tested whether NA up states showed changes over time in their synchronization with PFC electrical activity with *in vivo* intracellular recordings from NA core neurons acquired simultaneously with PFC LFPs in anesthetized adult male Sprague-Dawley rats (n = 13). NA neurons exhibited spontaneous transitions between a down state of −82.3±4.2 mV and up states of −54.5±2.6 mV (Figure 5*A*). NA up states are known to be strongly correlated with hippocampal LFPs but only weakly correlated with PFC fields when correlation is analyzed over long recording epochs [25]. To explore PFC-NA synchrony with a better temporal resolution (to determine whether brief variations can exist), we assessed cross-covariance of membrane potential values in NA neurons with LFPs in the PFC using a ±200 ms sliding window (Figure 5*B*). The covariance peaks typically corresponded to state transitions (Figure 5*C*). Selecting cross-covariance analyses for only the times of transition to the up state in NA MSNs revealed that not all state transitions co-varied with PFC LFPs (Figure 5*D*). In most NA neurons (8 of 10) the peak values of state transition-triggered cross covariance, as well as the value at zero lag, were significantly higher than cross covariance with randomized versions of the field potential (p<0.01; Figure 5*E*; the randomization was done conservatively, preserving the near-1 Hz oscillation characteristic of anesthetized recordings). Thus, NA up states can rapidly change their synchronization with PFC activity.

## Discussion

Multichannel recordings from awake, freely moving rats revealed that the synchrony among different frequency components of electrical activity in the PFC, NA and hippocampus can change according to the behavioral condition. During spatial exploration, theta rhythms in the hippocampus were synchronized with similar frequency peaks in the PFC and NA. During lever pressing for reward, PFC units became transiently active along with a brief increase in delta activity. NA recordings revealed that the NA core could lose its correlation with hippocampal theta activity (which persisted during the instrumental task) and acquire a synchronization with delta activity and low frequency events in the PFC. This switch may be interpreted as a brief activation of the PFC being able to drive active states in the NA and override the hippocampal influence. *In vivo* intracellular recordings tested this possibility and indeed showed that brief PFC activation can sometimes drive NA up states. Thus, the interplay among these structures critical for goal-directed behavior appears to be complex, and their impact on an output that may serve channeling their information into behavior can vary depending on the activity levels of the PFC.

We tested spatial exploration and lever pressing for sucrose in separate behavioral epochs within the same recording session. Although this approach is not identical to the exploration/exploitation tradeoff in the reinforcement learning framework where agents must constantly decide to utilize known sources of rewards or explore for better options [4], basic elements of the exploration and exploitation behaviors are likely to be represented in our experimental setting. Rats are choosing to explore or to lever press in the respective session phases rather than to rest, groom, or perform some other behavior that has value to the animal. Furthermore, we have taken care to avoid overtraining the animals so as to reduce the likelihood that responses are driven by habit-related neural circuits. Thus, the instrumental component in the recording session is likely to engage goal-directed processes [32] that are relevant to the exploitation of known sources of reward; the link of the spatial exploration component in our experiments to the exploration of possible new sources of reward is less apparent.

A long standing hypothesis is that the VH can “gate” cortical throughput in the NA [12]. Here we showed that NA up states can become transiently synchronized with PFC activity, indicating NA neurons could also be gated by PFC inputs. Thus, as the synchronization of NA MSN down and up states with PFC activity can change from one event to the next, slight variations in PFC activity could affect the membrane potential of NA MSNs. As a consequence, epochs with increased PFC firing such as during reward-seeking behavior could drive NA core cell firing by virtue of the tight control of NA MSN membrane potential by PFC bursts.

A remarkable finding is the presence of delta oscillations and slow events in the PFC and NA during the instrumental behavior. Although this can be seen as a slow deflection during the interval between the cue and the reward, low frequency events are detected in FFT analyses of 20 second-epochs around the lever press (Figure 3), suggesting that such delta activity is an important component of responses and may be present throughout the recording. It is unlikely that the slow deflection is due to a nonspecific factor such as movement, as it was not observed in electrodes located in the NA shell that are less than 1 mm away from the NA core wires that did show the delta component. Delta oscillations have been typically associated with slow-wave sleep [33] and may contribute to the consolidation of memory traces acquired during the state of wakefulness [34]. However, evidence also supports a role for delta activity in awake animals. For example, low frequency components in monkey motor cortex can carry information about parameters of voluntary arm movement [35], and such components have been reported in the dorsal striatum of rats during a procedural task [24]. Furthermore, delta oscillations in humans increase significantly while performing the Wisconsin Card Sorting Test [36], which typically activates the PFC. In the NA, delta oscillations are highest in amplitude during the awake quiet state or during grooming [37]. This pattern of activity is therefore likely to reflect internal activity states in the brain regions where the electrodes were located.

During the instrumental behavior component, the NA core, but not the shell, became synchronized with the PFC. Although the sample from shell sites is not large enough to be conclusive, we did not detect activity in the delta band in this region, even though wires from the same array located in the core did. This is important in highlighting that delta events are local and not due to volume conduction. The transient suppression of NA theta during the instrumental behavior despite persisting hippocampal theta also indicates that theta rhythms in both structures can be dissociated and argues against volume conduction. The core-shell difference in delta events during the task may allow some speculation that shell and core regions respond differently to medial PFC activation, and may have different roles in response selection. This possibility is consistent with reports that lesioning the NA core, but not shell, impairs shifting from one strategy to another [38], suggesting that the NA core facilitates acquisition and maintenance of novel behavioral strategies, and this role may depend on inputs from the prelimbic region of the medial PFC.

The integration of information in the NA does depend on the behavioral state of the animal. It is therefore likely that during behavioral conditions in which the VH is strongly active (i.e., exploration using spatial/contextual cues), the NA follows hippocampal commands and may serve to gate other cortical inputs [12]. Our *in vivo* data from anesthetized rats reveal that strong PFC activation with a bursty pattern similar to what has been reported in behaving rodents [20], [21] can drive persistent depolarizations in the NA [39]. Therefore, the NA could be envisioned as a critical relay station or switchboard that may determine the most appropriate behavioral output to the context and goals by selecting inputs that drive its activity according to the behavioral condition. The term switchboard is used here to represent a feature in the integration of inputs and intrinsic activity that may allow strong PFC activity to overcome the normally tight following of hippocampal inputs typically observed in the NA. Whether this is a feature of intrinsic NA circuits or driven by specific sets of afferents remains to be determined. A tight response to VH afferents during VH-dependent behaviors and to the PFC during PFC-dependent behaviors could ensure that the relevant sets of commands are transferred through the ventral basal ganglia loops whether the animal is engaged in spatial exploration or actively seeking reward. Our data show neural associations between essential structures for these functions during either condition, and highlight the NA core as a critical relay station that becomes engaged during reward-seeking behavior. The precise component of the instrumental task associated with the PFC-NA correlation cannot be discerned from our data. However, the behavioral conditions in which such synchrony was not evident included similar spatial information and locomotor levels, suggesting that these elements are not associated with the PFC-NA correlation. As the correlations are maximal during the lever press and approach to the reward well, they are likely associated with reward seeking. Unveiling how those states are achieved will certainly advance our understanding of an essential aspect guiding behavior and may unlock possible avenues to deal with conditions in which that balance becomes impaired such as neuropsychiatric disorders and drug addiction. Perseveration is a common observation in frontal lobe deficits and certain disorders that affect the PFC, such as schizophrenia [40]. It is possible that a PFC that fails to activate when required, as proposed for schizophrenia [41], will not allow its target neurons in the NA to select the appropriate outcome, causing acquired response patterns to persist. Thus, subcortical integration of corticolimbic inputs may have a strong impact on response selection.

## Supporting Information

Figure S1 Most NA neurons (52%) exhibited an increase in firing following lever press (type reinforcement-excitation (RFe); n = 31; A), 13% exhibited a decrease (type reinforcement-inhibition (RFi); n = 8; B); and 10% exhibited increases in firing rate preceding the lever press (i.e., between the cue and lever press; type pre-response (PR); n = 6; C). A small number (7%) of neurons exhibited a dual response (type PR+RF; n = 4). The remaining 18% neurons did not change firing during the reinforced response (non-phasic cells; n = 11). PFC neurons exhibited similar response patterns, as reported previously [22], [38]; they could also be classified as PR (15%; n = 5), RFe (32%; n = 11), RFi (32%; n = 11) or PR+RF (9%; n = 3). The remaining four PFC neurons (12%) did not show any change in firing rate. In the VH, almost half of the neurons (53%; n = 8) exhibited the RFe pattern and 47% (n = 7) of the neurons did not show any change in firing rate.
(5.76 MB TIF)

Figure S2 Histological confirmation of microelectrode tracks. Representative coronal Nissl-stained sections were used to identify electrode tracks and recording sites in the PFC, NA, and VH. The squared areas are zoomed and enlarged horizontally to illustrate the end of electrode tracks (arrowheads). Numbers indicate the distance to bregma in mm. Rightmost panels show histologically identified recording sites across all animals.
(2.59 MB TIF)

Table S1 Different types of phasic and non-phasic neurons in NA, PFC, and VH.
(0.03 MB DOC)
