# Supplementary figures and images for: The Nucleus Accumbens: A Switchboard for Goal-Directed Behaviors
Source: PLoS One. 2009 Apr 7;4(4):e5062. doi: 10.1371/journal.pone.0005062 (PMC2663037; doi:10.1371/journal.pone.0005062)

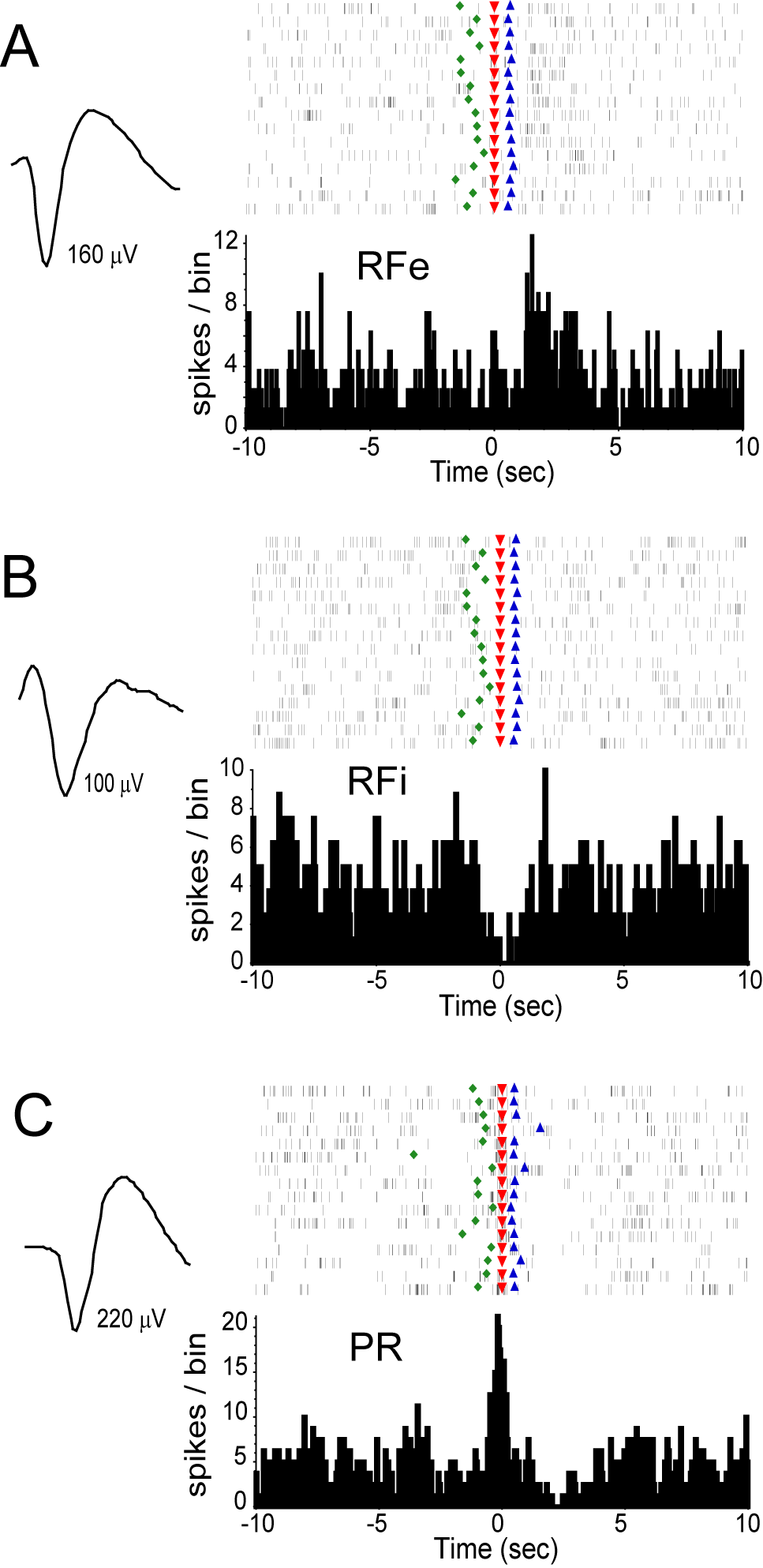

Supplement: Figure S1 — Most NA neurons (52%) exhibited an increase in firing following lever press (type reinforcement-excitation (RFe); n = 31; A), 13% exhibited a decrease (type reinforcement-inhibition (RFi); n = 8; B); and 10% exhibited increases in firing rate preceding the lever press (i.e., between the cue and lever press; type pre-response (PR); n = 6; C). A small number (7%) of neurons exhibited a dual response (type PR+RF; n = 4). The remaining 18% neurons did not change firing during the reinforced response (non-phasic cells; n = 11). PFC neurons exhibited similar response patterns, as reported previously [22], [38]; they could also be classified as PR (15%; n = 5), RFe (32%; n = 11), RFi (32%; n = 11) or PR+RF (9%; n = 3). The remaining four PFC neurons (12%) did not show any change in firing rate. In the VH, almost half of the neurons (53%; n = 8) exhibited the RFe pattern and 47% (n = 7) of the neurons did not show any change in firing rate. (5.76 MB TIF) [file pone.0005062.s001.tif]

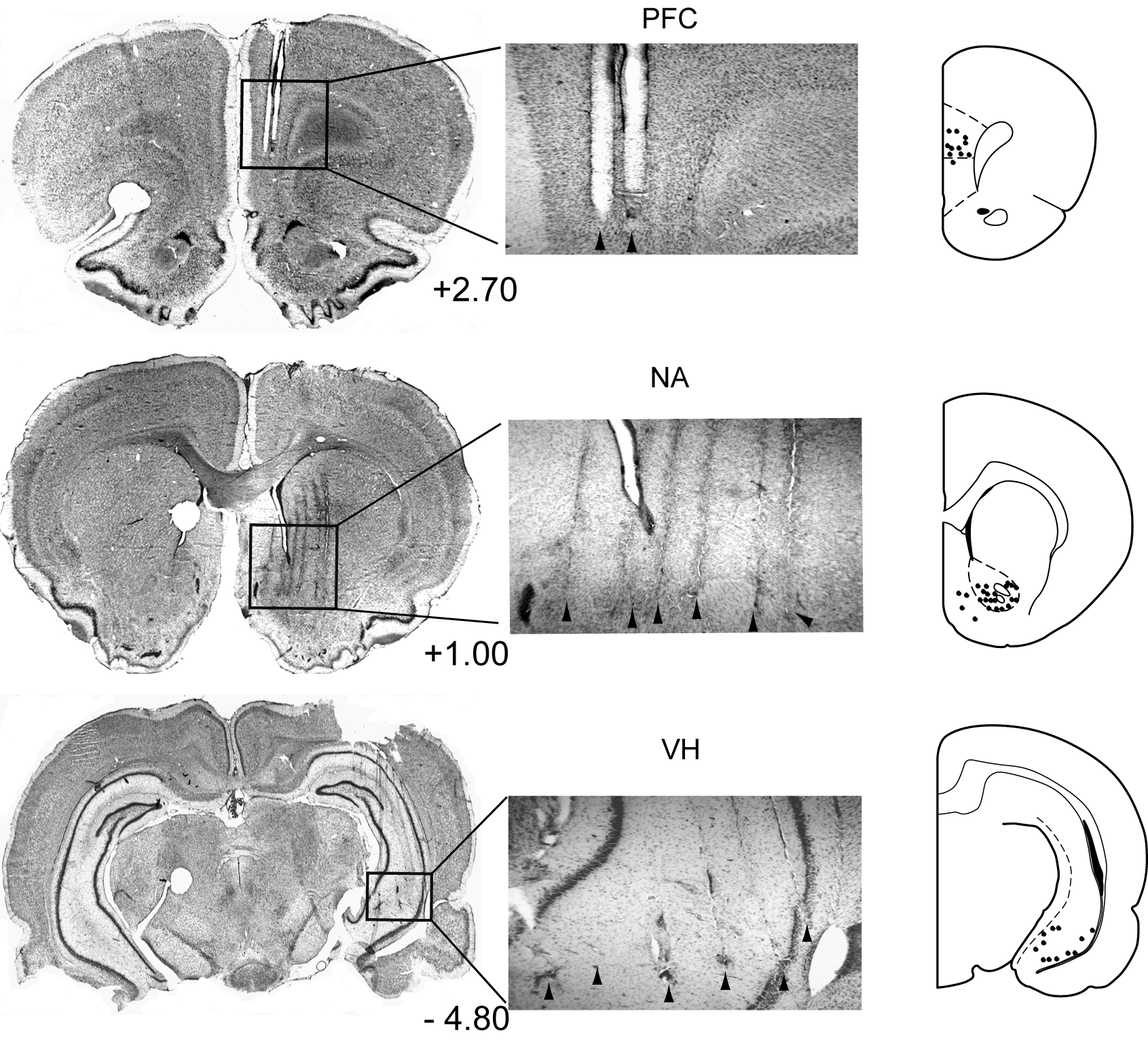

Supplement: Figure S2 — Histological confirmation of microelectrode tracks. Representative coronal Nissl-stained sections were used to identify electrode tracks and recording sites in the PFC, NA, and VH. The squared areas are zoomed and enlarged horizontally to illustrate the end of electrode tracks (arrowheads). Numbers indicate the distance to bregma in mm. Rightmost panels show histologically identified recording sites across all animals. (2.59 MB TIF) [file pone.0005062.s002.tif]
