# Supplementary material for: The Nucleus Accumbens: A Switchboard for Goal-Directed Behaviors
Source: PLoS One. 2009 Apr 7;4(4):e5062. doi: 10.1371/journal.pone.0005062 (PMC2663037; doi:10.1371/journal.pone.0005062)
Supplement: Table S1 — Different types of phasic and non-phasic neurons in NA, PFC, and VH. (0.03 MB DOC) [file pone.0005062.s003.doc]

**Table S1**. Different types of phasic and non-phasic neurons in NA, PFC and VH.

|  | RFe | RFi | PR | PR+RF | NR | Total |
| --- | --- | --- | --- | --- | --- | --- |
| NA | 31 (52%) | 8 (13%) | 6 (10%) | 4 (7%) | 11 (18%) | 60 |
| PFC | 11 (32%) | 11 (32%) | 5 (15%) | 3 (9%) | 4 (12%) | 34 |
| VH | 8 (53%) | 0 | 0 | 0 | 7 (47%) | 15 |
